# Gasdermins: multifunctional effectors of membrane permeabilization across cellular compartments

**DOI:** 10.1111/febs.70215

**Published:** 2025-08-10

**Authors:** Eleonora Margheritis, Nadine Gehle, Katia Cosentino

**Affiliations:** ^1^ Department of Biology/Chemistry and Center for Cellular Nanoanalytics (CellNanOs) University of Osnabrück Germany; ^2^ Department of Biomedical, Metabolic and Neural Sciences UNIMORE‐University of Modena and Reggio Emilia Italy

**Keywords:** gasdermins, inflammation and cancer, organelle membrane alteration, pore formation, pyroptosis regulation

## Abstract

Members of the gasdermin (GSDM) family are pore‐forming proteins primarily known for executing inflammatory cell death known as pyroptosis. GSDM‐mediated pore formation at the plasma membrane (PM) facilitates the selective secretion of immunomodulatory proteins and nonselective ionic fluxes during pyroptotic signaling. Recent findings suggest that GSDMs also modulate intracellular processes by associating with and altering membranes in various organelles, including mitochondria, lysosomes, endoplasmic reticulum (ER), and the nucleus. These activities may trigger alternative signaling pathways that do not necessarily involve PM perforation. In this review, we explore the diverse mechanisms of GSDM association across organelle membranes and discuss the physiological and pathological implications of GSDM‐induced membrane integrity alteration.

## Graphical abstract

Gasdermins (GSDMs) are pore‐forming proteins best known for driving pyroptosis through plasma membrane (PM) permeabilization. Beyond triggering inflammatory cell death, GSDMs can also associate with organelle membranes, including mitochondria, lysosomes, ER, and nucleus, where they modulate membrane integrity and cellular signaling. These nonlytic functions suggest broader physiological and pathological roles. This review explores GSDM targeting across subcellular membranes and its implications in infection and cancer.

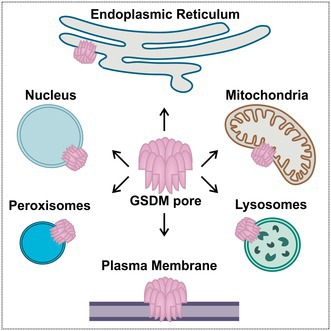

**Abbreviations**
AFM: atomic force microscopy
AKI: acute kidney injury
Apt‐OMVs: aptamer‐conjugated outer membrane vesicles
ARDS: acute respiratory distress syndrome
ASC: apoptosis‐associated speck‐like protein containing a caspase recruitment domain
ASM: acid sphingomyelinase
Ca^2+^
: calcium
cGAS: cyclic GMP‐AMP synthase
CL: cardiolipin
Crls1: CL synthase 1
CRS: cytokine release syndrome
cryo‐EM: cryo‐electron microscopy
CTD: C‐terminal domain
Cytc: cytochrome c
DAMPs: danger‐associated molecular patterns
DIC: disseminated intravascular coagulation
eCIRP: extracellular cold‐inducible RNA binding protein
eIF2α: eukaryotic translation initiation factor 2 alpha
ER: endoplasmic reticulum
ESCRT‐III: endosomal sorting complexes for transport‐III
EV71: enterovirus 71
FADD: Fas‐associated death domain
FL: full length
GSDMs: gasdermins
GZM: granzyme
HMGB1: high mobility group box 1
HSP75/HSP90: heat shock protein 75/90
IBD: inflammatory bowel disease
IEC: intestinal epithelial cells
IFNs: interferons
IL‐18: interleukin‐18
IL‐1β: interleukin‐1β
IRE‐1α: inositol‐requiring enzyme 1α
IRF2: interferon regulatory factor 2
K^+^
: potassium
LDH: lactate dehydrogenase
LPS: lipopolysaccharide
MAPL: mitochondrial anchored protein ligase
MD: molecular dynamics
MDVs: mitochondrial‐derived vesicles
MHCII: major histocompatibility complex class II
MLKL: mixed lineage kinase domain‐like
MLROs: mitochondria‐lysosome‐related organelle
MOMP: mitochondrial outer membrane permeabilization
mtDNA: mitochondrial DNA
mtROS: mitochondrial reactive oxygen species
NINJ1: ninjurin‐1
NLRP3: nod‐like receptor (NLR) family pyrin domain (PYD)‐containing 3
NP: nanoparticle
NSA: necrosulfonamide
NSCLC: non‑small cell lung cancer
NTD: N‐terminal domain
OSCC: oral squamous cell carcinoma
OxPaPC: oxidized 1‐palmitoyl‐2‐arachidonoyl‐sn‐glycero‐3‐phosphocholine
PA: phosphatidic acid
PA: prosapogenin A
PAMPs: pathogen‐associated molecular patterns
PARP‐1: poly [ADP‐ribose] polymerase 1
PDAC: pancreatic ductal adenocarcinoma
PE: phosphatidylethanolamine
Pi: phosphatidylinositol
Pi3P: phosphatidylinositol 3‐phosphate
Pi4P: phosphatidylinositol 4‐phosphate/PtdIns(4)P
PIP_2_
: phosphatidylinositol 4,5‐bisphosphate/PtdIns(4,5)P2/Pi(4,5)P_2_
PIP_3_
: phosphatidylinositol (3,4,5)‐trisphosphate/PtdIns(3,4,5)P3/Pi(3,4,5)P_3_
PIPs: phosphatidylinositol phosphates
PITs: pore‐induced intracellular traps
Plscr3: phospholipid scramblase 3
PM: plasma membrane
PS: phosphatidylserine
PTMs: post‐translational modifications
RIPK1/RIPK3: receptor‐interacting serine‐threonine protein kinase 1/3
sEVs: small extracellular vesicles
Smac/DIABLO: second mitochondria‐derived activator of caspase/direct inhibitor of apoptosis‐binding protein with low pI
SS: systemic sclerosis
STING: stimulator of interferon genes
TAK1: transforming growth factor‐β‐activated kinase 1
TAMs: tumor‐associated macrophages
TGFβ: transforming growth factor beta
TIME: tumor immune microenvironment
TMEM16F: transmembrane protein 16F
TNF: tumor necrosis factor
TNM: tumor‐node‐metastasis
Tom20/Tom70: translocase of outer mitochondrial membrane protein 20/70
Trap1: tumor necrosis factor receptor‐associated protein 1
YBX1: Y‐box‐binding protein 1
ZDHHC5: zinc finger DHHC‐type palmitoyltransferase 5
ZDHHC9: zinc finger DHHC‐type palmitoyltransferase 9

## Introduction

Gasdermins (GSDMs) are a family of pore‐forming proteins mediating pyroptosis, a form of inflammatory cell death involved in key biological and pathological processes, including antimicrobial responses, mitochondrial homeostasis, inflammation, and cancer [1].

Evolutionarily conserved from bacteria (with over 50 homologs) to mammals, GSDMs are also present in fungi and viruses, underscoring their ancient role in membrane remodeling [2, 3, 4]. In humans, the GSDM family includes six members: GSDMA, GSDMB, GSDMC, GSDMD, GSDME (also known as DFNA5), and PJVK (also referred to as DFNB59 or GSDMF), with four isoforms identified for GSDMB [5]. In mice, the family is further diversified, with 10 functional variants (GSDMA1‐3, GSDMC1‐4, GSDMD, GSDME, and PJVK) [1, 6, 7].

The name ‘gasdermin’ originates from the predominant expression of these proteins in the gastrointestinal tract and dermis. However, under normal conditions, GSDMs are broadly expressed across a variety of tissues and cell types, with particularly high levels in immune and epithelial cells. Their expression can increase further in response to immune activation, inflammation, or cellular stress, although the regulatory pathways and transcription factors involved remain largely unclear [8]. Notably, each GSDM family member exhibits a distinct tissue distribution. GSDMA is primarily found in the skin and gastrointestinal tract and is upregulated by transforming growth factor beta (TGFβ) [9], while GSDMB is present in the liver and gastrointestinal tract and shows additional lung expression mainly in cancerous tissues [10, 11]. GSDMB expression is independently upregulated by several interferons (IFNα, IFNβ, or IFNγ) and tumor necrosis factor (TNF) [12]. Unlike most GSDMs, GSDMC is absent from immune cells but is expressed in the skin, esophagus, small intestine, and colon [10]. GSDMD is predominantly found in myeloid cells and mucosal epithelial cells within the stomach, esophagus, and intestine, regulated in part by interferon regulatory factor 2 (IRF2) [10, 13, 14]. GSDME is more ubiquitously expressed in the brain, placenta, heart, kidney, intestine, ear, and muscle tissues, with its expression boosted by corticosteroids and forskolin [15, 16]. PJVK is highly expressed in the testis but is also found in other tissues, including the hair cells of the inner ear and various cells within the auditory system [17, 18, 19]. Interestingly, certain GSDMs, such as GSDME, and to some extent GSDMA, are epigenetically silenced by DNA methylation in cancer cells, a repression that can be reversed with DNA methyltransferase inhibitors [20, 21, 22, 23].

Structurally, all GSDMs (except PJVK) adopt an autoinhibited two‐domain architecture in their inactive cytosolic states consisting of a repressive C‐terminal domain (CTD) and an active, N‐terminal domain (NTD) with pore‐forming capacity. Upon cellular recognition of pathogen‐ or danger‐associated molecular patterns (PAMPs or DAMPs), or during cytotoxic lymphocyte attack, GSDMs are activated via proteolytic cleavage at the interdomain linker [24]. This liberates the functional NTD and facilitates its translocation to the plasma membrane (PM), where it assembles into pores via cooperative molecular interactions [1, 6, 25, 26]. These pores compromise PM integrity, leading to the release of cellular contents, including inflammatory cytokines [interleukin‐1β (IL‐1β)/interleukin‐18 (IL‐18)] into the extracellular space, thereby promoting inflammatory and immune responses by activating innate immunity [27].

The ability of GSDMs to execute pyroptosis places them at the heart of host defense against infection and cancer. Their activation triggers robust inflammatory responses, making them key effectors in innate immunity. Moreover, increasing evidence supports their role in potentiating anti‐tumor immunity through the promotion of immunogenic cell death [28, 29, 30]. However, when improperly regulated, pyroptosis can also contribute to detrimental inflammation, autoimmune pathologies, and cancer progression [31]. These dual roles highlight GSDMs as promising therapeutic targets: their controlled activation or inhibition could enable precise modulation of pyroptotic signaling, with far‐reaching implications for treating inflammatory diseases and enhancing cancer immunotherapy.

Beyond pyroptosis, GSDMs are emerging as multifunctional mediators involved in a spectrum of cell death pathways and organelle biology. Besides the PM, they can target various intracellular membranes, including those of mitochondria [32, 33, 34], endoplasmic reticulum (ER) [35, 36, 37], nucleus [38, 39, 40, 41, 42, 43, 44, 45], and lysosomes [46, 47]. Through these interactions, GSDMs influence organelle physiology and cellular stress responses in processes often unrelated to pyroptosis or involving alternative cell death pathways, such as apoptosis [36] and necroptosis [5, 48].

Given the expanding scope of GSDM functions and broader membrane targeting capabilities, there is growing interest in characterizing their roles in organelle dynamics and disease pathogenesis. This review explores the mechanisms by which GSDMs engage with diverse cellular membranes and assesses their broader physiological and pathological implications, particularly in inflammation and cancer biology.

## Mode of action of GSDMs

**Fig. 1:**
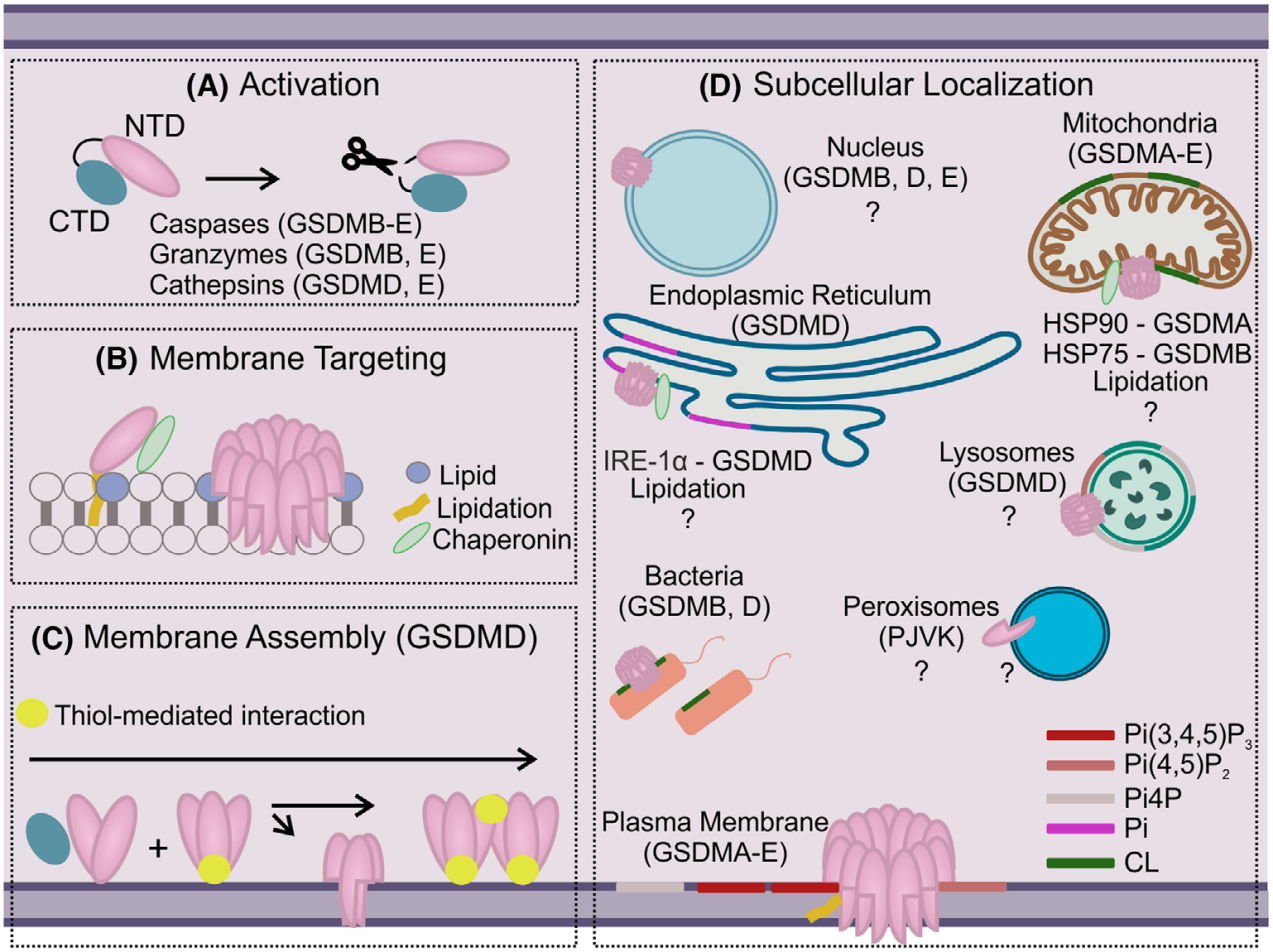
Mechanistic insights into the mode of action of gasdermins (GSDMs) at cellular membranes. (A) Protein activation occurs via proteolytic cleavage at the linker region connecting the N‐terminal domain (NTD) with the C‐terminal domain (CTD). These two domains maintain contact until they reach the membrane, where hydrophobic interactions with the lipid bilayer facilitate the release of the NTD from the CTD. (B) Membrane targeting can be mediated by different factors, including chaperone proteins, negatively charged lipids, and the lipidation of the protein. (C) Model of GSDMD pore assembly: At the membrane, NTDs of GSDMD assemble through a mechanism mediated by transient thiol interactions. This assembly process involves a first step of oligomerization, leading to the formation of basic dimeric/trimeric assembly units that may either proceed toward membrane insertion or undergo higher‐order oligomerization [26]. (D) Overview of the subcellular localization of GSDMs. Different GSDMs have been identified as capable of interacting not only with the plasma membrane (PM) but also with various organelles, including the endoplasmic reticulum (ER), nucleus, mitochondria, peroxisomes, lysosomes, and even directly with intracellular bacteria. Chaperon proteins, including heat shock proteins 75/90 (HSP75/HSP90) and the inositol‐requiring enzyme 1α (IRE‐1α), and the specificity of the membrane lipid composition, including Pi(4,5)P_2_ (Phosphatidylinositol 4,5‐bisphosphate), Pi(3,4,5)P_3_ (Phosphatidylinositol (3,4,5)‐trisphosphate), Pi4P (phosphatidylinositol 4‐phosphate), Pi (Phosphatidylinositol), and CL (cardiolipin), contribute to selective subcellular localization, pore formation, and stabilization of the pore structure.

Proteolytic cleavage by several cytosolic proteases releases the functional NTD from the regulatory CTD, leading to GSDM activation. After activation, GSDMs bind to cell membranes. This association is favored by both negatively charged lipids and protein lipidation [26, 49, 50, 51, 52, 53]. At the membrane, GSDMs undergo structural rearrangements that allow oligomerization and membrane insertion [49, 54, 55, 56, 57]. Importantly, lipid specificity contributes to selective subcellular localization, as well as to pore formation and stability. The sequence of events leading to GSDM pore formation is illustrated in Fig. 1 and described in the following sections.

### Molecular mechanisms of GSDM activation

GSDMs are cleaved by a diverse range of proteases, including inflammatory and apoptotic caspases, highlighting the intricate crosstalk between apoptosis and pyroptosis. Intriguingly, apoptotic caspases can either activate or suppress GSDM‐mediated cell death, serving as key regulators of inflammatory responses. Additionally, GSDMs are processed by granzymes and neutrophil‐derived serine proteases, such as cathepsin G and neutrophil elastase, reinforcing the role of GSDMs in immune defense.

Specifically, GSDMD, the best‐characterized effector of pyroptosis, is processed by caspase‐1 within the canonical inflammasome pathway, downstream of the assembly of an inflammasome platform composed of PAMPs/DAMPs sensors, such as nod‐like receptor (NLR) family pyrin domain (PYD)‐containing 3 (NLRP3), an adaptor protein (apoptosis‐associated speck‐like protein containing a caspase recruitment domain, ASC) and caspase‐1, which then undergoes auto cleavage for its activation and further processing of GSDMD [56]. Meanwhile, GSDMD can also be cleaved by caspase‐4/5 (11 in mice) through the noncanonical inflammasome pathway activated by intracellular lipopolysaccharide (LPS). GSDMD has also been shown to be processed by caspase‐8, cathepsin G, and neutrophil elastase [56, 58, 59, 60, 61].

Beyond GSDMD, GSDMA is cleaved by caspase‐1 in nonmammals, whereas in mammals only virulence factor SpeB has been identified so far [62]. GSDMB is processed by caspase‐1, as well as apoptotic caspase‐3, caspase‐6, and caspase‐7. In addition, granzyme A (GZMA), neutrophil elastase, and Derp3 all process GSDMB, suggesting a role in both immune defense and cell death regulation [63]. GSDMC is primarily processed by caspase‐8, linking its activation to extrinsic apoptotic signaling [64]. GSDME, another key player in the intersection of apoptosis and pyroptosis, is cleaved in mammals by apoptotic caspase‐3, granzyme B (GZMB), and cathepsins, demonstrating its involvement in both inflammatory and noninflammatory cell death pathways [15, 28, 65]. Notably, it is the only GSDM present in teleosts, where it exhibits a broader cleavage profile, being activated not only by apoptotic caspase‐3 and caspase‐7, but also by pyroptotic caspase‐1, which shows the highest efficiency [66]. No processing enzyme has yet been identified for PJVK, leaving its functional activation mechanism an open question.

In addition to these canonical cleavage events, some GSDMs undergo alternative processing at noncanonical sites within the NTD. This may result in truncated fragments that are unable to form functional pores, effectively blocking pyroptosis. Intriguingly, apoptotic caspase‐3 and caspase‐7, typically associated with noninflammatory apoptotic cell death, have been shown to inhibit GSDMD through cleavage at a specific site in the NTD, thereby causing inactivation of its pore‐forming activity [67]. This may serve as a regulatory checkpoint that prevents excessive inflammation. Moreover, several pathogens, including Enterovirus 71 (EV71) and SARS‐CoV‐2, exploit this mechanism to evade GSDM‐mediated immune responses, further emphasizing the importance of GSDMs in host defense [68, 69, 70].

Furthermore, cleavage‐independent activation of both GSDMD and GSDME has also been proposed under specific conditions. This alternative activation is thought to be driven by conformational relaxations induced by bulky post‐translational modifications (PTMs) that weaken the strong autoinhibition exerted by the CTD and promote oligomerization of the full‐length (FL) protein [51, 71]. Although the resulting pore formation is less efficient than the canonical cleavage‐dependent pathway, it may still represent a meaningful cellular response to damage signals, particularly when caspase activity is impaired. Structurally, this mechanism suggests that GSDM autoinhibition can be relieved by conformational changes alone, independently of proteolytic cleavage. This opens the possibility of designing small molecules that destabilize the interface between the autoinhibitory and active domains to promote activation of GSDMs. Such strategies could hold therapeutic potential for pyroptosis activation in pathological settings, particularly in contexts where a milder inflammatory response is desired. However, additional structural and functional studies, including high‐resolution structures of FL GSDMs with or mimicking relevant PTMs and cell‐based experiments expressing PTM‐mimetic constructs in caspase‐deficient backgrounds, are required to validate and further characterize this backup activation pathway.

### Gasdermin membrane targeting: lipids as drivers of GSDM cellular localization

Lipid membrane composition precisely determines the ability of GSDMs to associate with cellular membranes, governing their binding and membrane insertion (Fig. 1) [49, 54, 72].

GSDMA (and GSDMA3), GSDMD, and GSDME exhibit a strong affinity for phosphorylated phosphatidylinositols (PIPs), particularly bisphosphorylated PtdIns(4,5)P2 (PIP_2_), which is crucial for biological processes, such as membrane trafficking and cytoskeletal dynamics [15, 26, 49, 54, 72]. Unlike the other GSDMs, the affinity of GSDMB for PIPs is more debated [73, 74]. PIPs are primarily localized to the inner leaflet of the PM and to the cytosolic leaflet of various intracellular membrane compartments. PIPs compositional asymmetry at the PM contributes to the selective intracellular functionality of GSDMs, preventing them from binding and permeabilizing cells from outside [49, 55]. To reinforce this concept, GSDMA3 and GSDMD also bind with weaker affinity to phosphatidic acid (PA) and phosphatidylserine (PS), both found in the inner PM leaflet, while failing to effectively interact with lipids present in both PM leaflets, such as phosphatidylethanolamine (PE) and cholesterol [49, 55, 75]. The reduced membrane association due to cholesterol is likely a consequence of the formation of organized domains and decreased membrane fluidity, as shown for the GSDM‐structurally similar perforins [76]. In support of the critical role of PIPs in targeting GSDMs to the PM, pathogens like *Mycobacterium tuberculosis* dephosphorylate monophosphorylated PtdIns(4)P (Pi4P) and PIP_2_ at the PM to inhibit GSDMD‐NTD membrane localization and prevent pyroptosis, thus evading host immunity and ensuring intracellular survival [77]. Furthermore, mature IL‐1β localizes to PIP_2_‐enriched regions of the PM, in the proximity of GSDMD pores, likely facilitating its pore‐mediated secretion [78]. Notably, the cytosolic leaflets of Golgi, early and recycling endosomes, and lysosomes all contain PIPs to which GSDMs have affinity, suggesting GSDM‐NTD localization to these organelles [54, 79, 80].

Intriguingly, cardiolipin (CL), exclusively found in bacterial and mitochondrial membranes, serves as a universal anchor for GSDMs [15, 32, 34, 49, 54, 73, 81, 82]. The strong affinity for CL has prompted a number of studies to disclose a new role of GSDMs in permeabilizing mitochondria and orchestrating mitochondrial signaling pathways. GSDM recruitment to mitochondria is facilitated by CL exposure to the mitochondrial outer membrane, mediated by CL synthase 1 (Crls1) and phospholipid scramblase 3 (Plscr3) during pyroptosis [32]. CL is also an integral component of Gram‐negative bacterial membranes, suggesting a direct bacterial killing function for GSDMs [54, 73, 83, 84].

### Gasdermin membrane targeting: protein lipidation

Early studies on GSDMD highlighted the key role of specific cysteine residues, particularly Cys191 (Cys192 in mice) in pore formation [54, 85]. Targeting Cys191 with GSDMD inhibitors, such as disulfiram or necrosulfonamide (NSA), prevents the assembly of high‐order GSDMD oligomers and inhibits pyroptosis [85, 86]. The initial interpretation of these data suggested that intra‐ or inter‐disulfide bridges might hold GSDMD oligomers together [54]. However, cryo‐electron microscopy (cryo‐EM) studies of GSDMD pore structure did not support this idea, as the cysteines were not close enough to form disulfide bonds [57]. The puzzling role of Cys191 was clarified more recently, thanks to a number of studies indicating that palmitoylation at this residue mediates GSDMD localization at the PM [26, 50, 51, 52, 53, 87]. This suggests that Cys191 influences oligomerization indirectly by playing a critical role in the initial membrane recruitment step. Located in a disordered loop, this cysteine residue is highly exposed to the cytosolic aqueous environment, making it easily accessible for palmitoylation [88]. This modification is catalyzed by the palmitoyl acyltransferases: Zinc finger DHHC‐type palmitoyltransferase 5 (ZDHHC5) and zinc finger DHHC‐type palmitoyltransferase 9 (ZDHHC9), whose expression is regulated by inflammasome activation and reactive oxygen species (ROS) [51].

Interestingly, in our recent study, we found that the C191A (or C192A) mutation does not completely abolish PM association, as long as negatively charged lipids are present. This emphasizes the synergistic role of palmitoylation and the membrane environment in promoting GSDMD membrane association [26].

Beyond GSDMD, other GSDMs (A, B, C, and E) are also palmitoylated at the NTD [51], while GSDME additionally undergoes palmitoylation at the CTD [89], suggesting shared regulatory mechanisms. However, the specific cysteine residues involved and the enzymes responsible for these modifications remain to be fully identified. It is also unclear whether palmitoylation, or other potential lipidation modifications, directly influence GSDM targeting to specific cellular membranes. To date, GSDM organelle targeting has primarily been attributed to lipids or chaperone proteins (Fig. 1). A systematic investigation into GSDMs lipidation modifications is still lacking but is essential for understanding their roles in membrane targeting and subcellular localization.

### Crucial role of cysteine residues in supporting oligomerization

The molecular mechanism underlying membrane assembly has been elucidated in PM‐mimicking membranes for GSDMD (Fig. 1) [26]. At the membrane, GSDMD assembles into dimeric and trimeric units, which then further oligomerize into higher‐order oligomers. Notably, membrane insertion follows oligomerization, with dimers and trimers being the minimal assembly units capable of inserting into the membrane [26]. This process is exclusively dependent on Cys192, Cys39, and Cys57 (Cys191/Cys38/Cys56 in humans), likely mediated by transient thiol interactions (Fig. 1). Intriguingly, any of these cysteine residues is sufficient for the formation of dimers and trimers, but all three are required for the formation of higher‐order oligomers [26]. Whether this mechanism is applicable to all GSDMs remains to be investigated. The critical role of cysteine residues in GSDM assembly is instrumental for the design of therapeutic inhibitors. Indeed, several small molecules inhibiting GSDMD assembly have been identified, specifically targeting Cys192 (Cys191 in human) [85, 86, 87, 90] and Cys57 (Cys56 in human) [91].

### GSDM pore structure and functionality

Cryo‐EM structures of GSDMA3, GSDMB, and GSDMD pores have revealed drastic conformational changes as these proteins transition from their soluble state to membrane perforation [49, 57, 88, 92, 93, 94]. All three structures exhibit the formation of a ‘pre‐pore’, suggesting that a full ring‐shaped structure can assemble on the membrane before undergoing the conformational changes necessary for membrane insertion [57, 92, 93]. This supports a concerted mechanism of assembly [7, 26]. High‐resolution atomic force microscopy (AFM) studies in artificial membranes further confirm this mechanism, showing that GSDMA3 oligomeric assemblies remain attached and mobile in a pre‐pore state before fully perforating the membrane [95].

The pore structure consists of elongated β‐hairpins that assemble a β‐barrel channel with slight differences among the various members of the family. The GSDMB isoform 1 pore is the smallest, featuring a 24‐fold symmetry, an inner diameter of 15 nm, and an outer diameter of 25 nm [92] while isoform 4 forms a 27‐fold pore with 16/27 nm inner/outer diameters [94]. The GSDMA3 pore exhibits a 27‐fold symmetry, with inner and outer diameters of 18 and 28 nm, respectively [93]. Human GSDMD forms a 33‐fold symmetry pore of 21 nm inner and 31 nm outer diameters [57].

While cryo‐EM studies have primarily captured fully assembled ring‐shaped GSDM pores, AFM characterization of GSDM pores in artificial membranes has revealed diverse oligomeric complexes, including arcs and slits, which can evolve into complete rings in both GSDMD [72] and GSDMA3 [95]. Recently, we have resolved GSDMD structures directly in the native PM of pyroptotic cells by super‐resolution microscopy and unveiled various macromolecular architectures, from small clusters to arc‐ and ring‐shaped GSDMD oligomers, with arcs and rings capable of membrane permeabilization [96].

The identification of physiologically heterogeneous structures raises the intriguing possibility that GSDM functionality, and consequently, the strength of the immune response is regulated not only by the number of pores but also by their size and geometry. A reduction in pore number or size, due to lower GSDM levels or reduced gene activity, may lead to a mild immune reaction that may persist without inducing immediate cell death [97, 98]. This regulation may occur early in pyroptosis or when GSDM activity is inhibited, allowing inflammation to continue without rapid cell destruction. Interestingly, certain GSDMB isoforms lack a structural component known as the belt motif, which is essential for stable pore formation. This suggests a built‐in regulatory mechanism in which these inactive GSDMB variants function as natural inhibitors, fine‐tuning pore formation and immune activation [99].

Additionally, it would be interesting to investigate whether the formation of small GSDMD oligomers in the early stage of pyroptosis [26, 96] may already be sufficient to permeabilize the PM. This could serve distinct biological purposes, such as facilitating the passage of small molecules like calcium ions (Ca^2+^) to activate downstream signaling pathways.

Notably, GSDMD pores function as selective channels, discriminating between molecules based not only on size but also on charge [57]. Acidic patches within the GSDMD pore conduit promote the preferential release of basic, positively charged, and neutral cargoes. Whether transport specificity varies among different members of the GSDM family remains to be determined, as well as whether pore size and shape differ when targeting distinct cellular membranes.

## GSDMs multifaceted function at cellular membranes

**Fig. 2:**
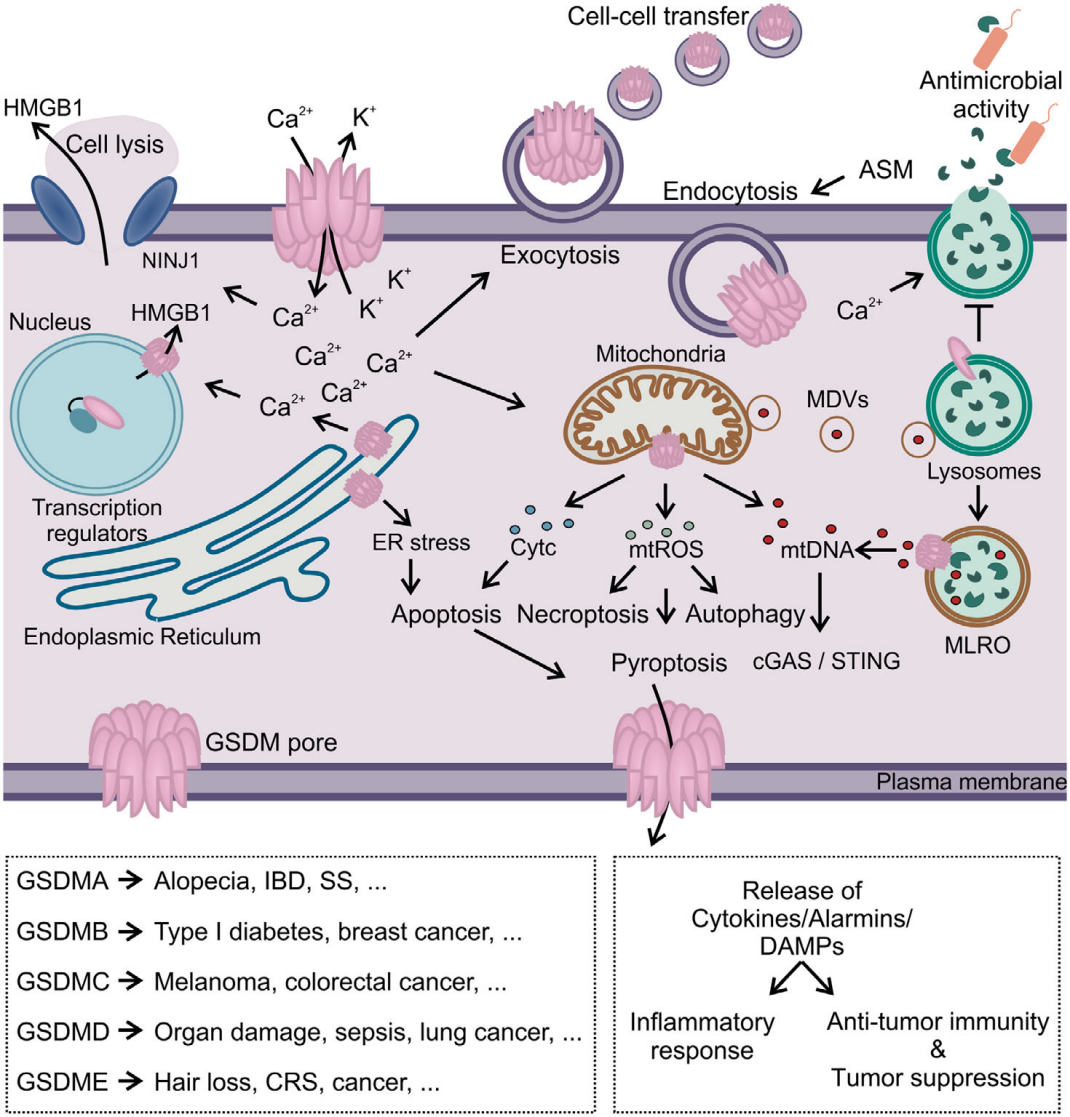
Physiological and pathological consequences of gasdermin (GSDM) membrane localization. Upon activation, GSDMs localize at different cellular membranes. At the plasma membrane (PM), GSDMs mediate the extracellular release of danger‐associated molecular patterns (DAMPs), immunomodulators and ion fluxes, including calcium (Ca^2+^) influx and potassium (K^+^) efflux, that orchestrate several cellular responses. The extracellular release of cytokines and alarmins through GSDM pores serves as a powerful trigger of inflammation and adaptive immunity and plays a significant role in activating anti‐tumor immunity for tumor suppression. Calcium intake exhibits a dual role: Intracellular calcium is important for the induction of lysosomal exocytosis thereby triggering the release of antimicrobial factors and acid sphingomyelinase (ASM) to activate the ASM‐mediated membrane repair mechanism counteracting cell death. Cytosolic calcium can also activate ninjurin‐1 (NINJ1) thereby triggering complete cell lysis. Subcellular localization of GSDMs is emerging as a significant initiator of organellar dysfunction and alterations. At the mitochondria, GSDM pores induce mitochondrial reactive oxygen species (mtROS) production and allow the release of mitochondria content, including cytochrome c (Cytc) and mitochondrial DNA (mtDNA), while inducing activation of different cell death pathways. At the endoplasmic reticulum (ER), GSDM pore formation mediates further calcium mobilization responsible for cytosolic translocation of nuclear High mobility group box 1 (HMGB1) and induces ER stress thereby initiating apoptosis in cancer cells. GSDM localization at the lysosomes has controversial effects of either inhibiting lysosomal maturation and exocytosis or inducing the cGAS‐STING pathway upon pore formation of mitochondria‐lysosome‐related organelle (MLROs) formed upon fusion of mitochondrial‐derived vesicles (MDVs) with lysosomes. At the nucleus, inactive GSDMs act as potent transcription regulators, while active proteins are responsible for HMGB1 secretion. Inflammatory pathways triggered by GSDM‐induced membrane alterations play a critical role in immune responses to infection and the activation of anti‐tumor immunity. However, excessive or chronic GSDM activation is linked to a range of inflammatory disorders, including alopecia, diabetes, inflammatory bowel disease (IBD), systemic sclerosis (SS), cytokine release syndrome (CRS), and tumor progression.

Beyond their well‐known role in pyroptosis through PM permeabilization, emerging evidence suggests that GSDMs contribute to diverse cellular processes by localizing to different subcellular membranes. These multifaceted functions and their physiological consequences are explored in the following section (Table 1 and Fig. 2).

**Table 1:** Gasdermin (GSDM) localization and function at different subcellular membranes.

GSDM family member	Targeted membranes	Functional outcome	References
GSDMA	PM	ATP release	[100]
	Mitochondria	Enhanced mtROS production and autophagy induction	[32, 101, 102, 103]
	Nucleus	Unknown	[45]
GSDMB	PM	ATP release	[100]
	Mitochondria	Enhanced mtROS production	[5]
	Nucleus	Upregulation of TGF‐ß1	[38, 39]
GSDMC	PM	ATP release	[100]
GSDMD	PM	ATP release Secretion of interleukins: IL‐1α, IL‐1ß, IL‐18, IL‐33 Secretion of eCIRP, Galectin‐1 Potassium efflux and subsequent inhibition of cGAS Calcium influx and NINJ activation	[49, 50, 57, 100, 104, 105, 106, 107, 108, 109, 110, 111, 112, 113, 114]
	Mitochondria	Enhanced mtROS production Release of mtDNA	[32, 34, 48, 115]
	Endosomes	Reduced endo‐lysosomal maturation	[46]
	Lysosomes	Membrane permeabilization Release of mtDNA from lysosomes	[46, 116, 117]
	Nucleus	Inhibition of DNA damage repair mechanisms Induction of expression of CIITA and MHCII to activate Tr1 T cells in the small intestine Activation of STAT5a to induce expression of CXCL1	[40, 42, 43]
	ER	Activation of ER stress and enhancing autophagy or apoptosis Calcium leakage from ER	[35, 36, 37, 118]
	Extracellular vesicles	IL‐1ß secretion Dissemination of GSDMD to neighboring cells to promote cell death	[119, 120]
GSDME	PM	ATP release Secretion of IL‐1α, IL‐1ß, IL‐18	[100, 121]
	Mitochondria	Enhanced mtROS production Release of mtDNA Enhanced immunogenic cell death	[32, 33, 34, 49, 82, 115, 122, 123, 124]
	Nucleus	Promotion of mucin expression by mediating entry of YBX1 into the nucleus Translocation of p65 into nucleus to progress skin inflammation	[41, 44]
PJVK	Peroxisomes	Proliferation of peroxisomes Degradation of damaged peroxisomes	[125, 126]

### GSDM localization at the plasma membrane

All GSDMs, except PJVK, have been demonstrated to permeabilize the PM in response to infection or dangerous stimuli [6]. GSDM pores at the PM serve as conduits for the unconventional secretion of bioactive molecules into the extracellular space to activate inflammatory responses [104, 121]. Moreover, they generate nonselective ion fluxes, including potassium efflux and calcium influx, thereby activating related downstream signaling pathways [127].

PM undergoes profound alterations following GSDM membrane targeting. The self‐assembly of GSDMs into PM pores contributes to cell flattening and is associated with the formation of pyroptotic bodies, also known as pore‐induced intracellular traps (PITs) when they retain captured bacteria. Pyroptotic bodies have a diameter of 1–5 μm and can also be engulfed by professional phagocytes during efferocytosis for maintaining tissue homeostasis [128, 129]. PM also generates nanometer‐sized extracellular vesicles containing GSDMD pores that serve as vehicles for facilitating information exchange among cells and propagating pyroptosis [119].

Furthermore, GSDMD‐induced membrane perforation generates a local concave curvature of the membrane toward the extracellular space in close proximity to the pore [57]. This curvature, along with calcium influx, facilitates the assembly of membrane repair machinery. Mammalian cells can repair small‐ to medium‐sized (10–100 nm) PM lesions through various mechanisms, including lysosomal acid sphingomyelinase (ASM)‐dependent endocytosis and endosomal sorting complexes for transport‐III (ESCRT‐III)‐mediated membrane shedding. Both of these processes have been shown to help limit membrane damage caused by GSDMD pores [130, 131, 132].

Notably, the PM lipid composition undergoes significant changes to regulate GSDM pore activity. This includes dynamic PIPs metabolic switches, which cells commonly used to control PM permeabilization by various proteins [133]. Local phosphoinositide metabolism, driven by calcium influx, determines the opening and closing of individual GSDMD pores [134]. Atomistic molecular dynamics (MD) simulations have shown that PIP_2_ and PIP_3_ play a crucial role in stabilizing the assembly of functional GSDMD ring structures [96, 135]. These lipids accumulate at the interface of adjacent GSDMD subunits, acting like a double‐sided tape to promote their interaction. Among these lipids, PIP_3_, enriched at the PM during the early stage of pyroptosis [96], demonstrates a stronger stabilizing effect than PIP_2_. It binds to five positively charged residues on human GSDMD (Lys43, Arg53, Lys55, and Arg153 on one subunit and Lys235 on the other subunit), reinforcing pore opening and preventing the collapse of intermediate pore structures. In line with this, depleting PIP_3_ reduces the formation of fully assembled ring structures as well as the size of GSDMD pores in cells [96].

Whether these mechanisms are common to all GSDMs remains to be investigated. Nevertheless, these insights open new avenues for regulating GSDM pore formation by manipulating PIP metabolism, offering a strategic approach to modulate pyroptosis in various diseases.

### Physiological consequences of GSDM localization at the plasma membrane

Although the full spectrum of biologically active molecules that may be released through GSDM pores remains underexplored, these pores primarily function as secretion pathways for alarmins and interleukins of the IL‐1 family [136, 137]. IL‐1β and IL‐18 secretion is modulated, upon inflammasome activation, by GSDMD and GSDME pores [104, 121]. GSDMD pores also play a dual role in the processing and secretion of IL‐1α: upon inflammasome activation, Ca^2+^ influx mediated by GSDMD pores results in the calpain‐dependent maturation of IL‐1α, which is subsequently secreted from the cell [105]. Additionally, IL‐33 has also been identified as being secreted through GSDMD pores in senescent hepatic stellate cells, thereby promoting obesity‐associated hepatocellular carcinoma [106] and following allergen exposure promoting allergic diseases [107]. Besides interleukins, GSDMD pores mediate the release of extracellular cold‐inducible RNA binding protein (eCIRP) from living macrophages, suggesting that targeting GSDMD could be a novel and potential therapeutic approach to inhibit eCIRP‐mediated inflammation in sepsis [108]. Galectin‐1 is also released through GSDMD pores as a consequence of cytosolic LPS sensing [109]. All GSDMs (except PJVK) also mediate early ATP release from macrophages. This process occurs before IL‐1β release and pyroptotic cell death, serving as a transient early danger signal that is independent of cell lysis and may occur through initial small pores [100].

GSDM pores at the PM also generate ionic fluxes that can impact cellular functions. Potassium efflux may have several cellular consequences, including activation of the NLRP3 inflammasome and inhibition of the cytosolic DNA sensor cyclic GMP‐AMP synthase (cGAS)‐dependent type I interferon responses, both of which enhance pyroptosis [110]. Cytosolic calcium increase has opposing effects on GSDM‐mediated cell death. At early stages of membrane damage, it activates membrane repair mechanisms that support cell survival. In contrast, at later stages, it activates calpain‐dependent cleavage of the cytoskeletal protein vimentin and, in turn, drives ninjurin‐1 (NINJ1) oligomerization at the PM, leading to complete cell lysis [111, 112, 113, 114]. NINJ1 oligomers create large lesions in the PM, allowing the release of DAMPs, such as high mobility group box 1 (HMGB1), lactate dehydrogenase (LDH), and other large proteins and protein complexes into the extracellular space.

Additionally, calcium influx activates transmembrane protein 16F (TMEM16F), a phospholipid scramblase that translocates phosphatidylserine (PS) from the inner to the outer leaflet of the PM, functioning as an ‘eat me’ signal [138, 139]. Finally, calcium mobilization induces the activation of cellular processes that help combat infection, including lysosomal exocytosis, which allows for the release of antimicrobial host proteins. These proteins can remain active in the extracellular environment to kill bacteria in the vicinity of cells undergoing pyroptosis [140]. It remains to be determined whether all GSDM pores allow calcium influx and whether additional calcium‐mediated cellular events occur during pyroptosis. While structural similarities between GSDMs support the hypothesis that pores formed by different GSDM family members likely lead to similar intracellular consequences, data directly supporting this hypothesis are scarce and warrant further investigation.

GSDM‐mediated PM pore formation is not always a death sentence. Although cell death has been assumed to be the main function of GSDM pores, increasing evidence suggests that these pores also have nonlytic functions. In this case, cells undergo a prolonged, controlled release of cytokines and alarmins in the absence of cell death. This state, called ‘hyperactivation’, occurs under certain conditions in neutrophils [141] and dendritic cells stimulated with oxidized 1‐palmitoyl‐2‐arachidonoyl‐sn‐glycero‐3‐phosphocholine (OxPaPC) [97]. This cellular state accounts for the activation of adaptive immunity and anti‐tumor immunity [98, 142] and underscores intriguing yet‐to‐be‐identified regulatory mechanisms of GSDM PM pore formation.

### GSDM localization at mitochondria

While mitochondria's primary function is energy production, cell differentiation, and cellular metabolism, they also play a crucial role in regulating cell death [143]. Mitochondrial outer membrane permeabilization (MOMP) is a key step in the intrinsic pathway of apoptosis and is primarily mediated by the Bcl‐2 family proteins BAX and BAK [144, 145].

GSDMs have also been observed to associate with mitochondria in various cell types in response to different stimuli, following an evolutionarily conserved mechanism [32, 33, 34, 48, 101, 115, 146]. Gain‐of‐function mutations in GSDMA3, as well as overexpression of a chimeric activable human GSDMA, have demonstrated that this protein preferentially targets mitochondria over the PM. This targeting occurs through interactions with the heat shock protein 90 (HSP90), the translocase of outer mitochondrial membrane protein 70 (Tom70) and the mitochondrial chaperone tumor necrosis factor receptor‐associated protein 1 (Trap1) [101, 102]. GSDMB‐NT colocalizes with Tom20 and HSP75/Trap1 on mitochondria [5]. For GSDMD and GSDME, mitochondrial targeting is primarily driven by their ability to bind oxidized CL [15, 32] (Fig. 1). Mitochondrial targeting of GSDMs results in permeabilization of mitochondrial membranes, likely preceding PM permeabilization [32, 34, 82, 101, 147]. Whether this process intersects with BAX/BAK pore formation, and how it does, may depend on the specific GSDM and cellular context [32, 148]. GSDME likely targets mitochondria following BAX/BAK‐mediated MOMP, as its activation requires apoptotic caspase‐3 [15, 34, 82]. Conversely, inflammasome‐generated active GSDMD has been proposed to permeabilize mitochondria to activate caspase‐3, thereby accelerating the apoptotic pathway [34]. Furthermore, mitochondrial ROS promote GSDMD oligomerization [149, 150], and it has been suggested that GSDMD pores at the mitochondria enhance ROS production in a positive feedback loop to accelerate pyroptosis (see following section) [32]. Altogether, this evidence raises important questions about the factors that regulate the preferential targeting of GSDMs to mitochondria versus the PM and the functional consequences of GSDM‐mediated mitochondrial permeabilization.

### Physiological consequences of GSDM localization at mitochondria

The localization of GSDMs at mitochondria has significant physiological consequences, influencing both cell survival and cell death pathways. GSDMs at mitochondria contribute to MOMP, resulting in the release of pro‐apoptotic factors, such as cytochrome c (Cytc) and second mitochondria‐derived activator of caspase/direct inhibitor of apoptosis‐binding protein with low pI (Smac/DIABLO). This disruption of mitochondrial integrity causes a loss of transmembrane potential, fragmentation of the mitochondrial network, mitophagy, and impaired oxidative phosphorylation (OXPHOS), ultimately leading to bioenergetic failure and metabolic shifts [5, 32, 33, 34, 82, 115]. Additionally, GSDM pores enhance the production of ROS, creating a positive feedback loop that further promotes GSDM oligomerization and accelerates pyroptosis [32]. ROS production also upregulates the palmitoyltransferases that induce S‐palmitoylation at GSDMD Cys191, thereby promoting its targeting to the PM [50, 51]. ROS are also emerging as key regulators of GSDM functionality at mitochondria, influencing cell death fate. Intracellular mitochondrial ROS generated by GSDMB and GSDMD promote a switch from pyroptosis to receptor‐interacting serine–threonine protein kinase 1 (RIPK1)/receptor‐interacting serine–threonine protein kinase 3 (RIPK3)/mixed lineage kinase domain‐like (MLKL)‐dependent necroptosis [5, 48]. GSDMA3 binding to mitochondria and the resulting ROS increase ultimately lead to autophagy [103]. Mitochondrial ROS is also both a cause and consequence of GSDME mitochondrial pore formation. Specifically, ROS production enhances caspase‐3 mediated activation of GSDME, which, in turn, further increases ROS levels and leads to complete mitochondrial dysfunction. GSDME activation then promotes PM permeabilization, shifting apoptosis to pyroptosis [34, 105, 115]. In cancer, GSDME‐mediated mitochondrial targeting and apoptosis‐to‐pyroptosis switch enhance immunogenic cell death, potentially influencing tumor suppression or immune evasion. This has been observed in melanoma cells [122], in ovarian cancer cells treatment with gambogic acid [123] and cells exposed to synthetic anti‐tumor cyclometalated Ir(III)‐lonidamine (LND) complexes [124]. Additionally, GSDM‐mediated mitochondrial damage induces the release of mitochondrial DNA (mtDNA) from the matrix and intermembrane space [32, 115], triggering inflammatory responses through activation of the cGAS‐stimulator of interferon genes (STING) pathway and type I interferon production [151] in endothelial cells [152] and cardiomyocytes [153]. It is unclear whether GSDMs can target the inner mitochondrial membrane directly or whether its rupture occurs as a secondary consequence of mitochondrial damage following GSDM association. Understanding how mtDNA is released upon GSDM‐mediated mitochondrial permeabilization requires further investigation.

### GSDM localization at endosomes and lysosomes

Direct evidence of GSDM interactions with endosomal and lysosomal membranes is limited but highly intriguing. In osteoclasts, upon progression of osteoclastogenesis, a gradual decrease in transforming growth factor‐β‐activated kinase 1 (TAK1) shifts the signaling pathway toward activation of RIPK1, which, in turn, triggers caspase‐8 activation in complex with the Fas‐associated death domain (FADD), leading to caspase‐3 activation. Caspase‐3 cleaves GSDMD to produce a nonlytic p20 fragment that localizes and oligomerizes on early endosomes. Notably, rather than forming pores, p20 GSDMD binds phosphatidylinositol 3‐phosphate (Pi3P) and sequesters it, thereby blocking its conversion, a critical step for endo‐lysosomal maturation and secretion. This activity inhibits lysosomal function and bone resorption, ultimately preventing bone loss and preserving bone homeostasis [46].

Lysosomal membrane permeabilization is a conserved feature of pyroptosis that precedes PM damage, resulting in the release of lysosomal contents, such as cathepsins, into the cytosol [65, 147, 154]. In anaplastic thyroid cancer, Prosapogenin A (PA) over‐acidifies lysosomes via vacuolar ATPase (V‐ATPase) activation, leading to cathepsin activation of caspase‐8 and, subsequently, caspase‐3 to mediate GSDME cleavage and pyroptotic cell death [65]. Lysosomes also serve as a recruiting platform for GSDM activation. During *Yersinia* infection, the bacterial effector protein YopJ inactivates TAK1 kinase, triggering FADD‐RIPK1‐caspase‐8 complex recruitment to the Rag regulator platform located on the cytosolic surface of lysosomes. The GTPase activity of the Rag regulator complex enables RIPK1 phosphorylation and caspase‐8 activation, which subsequently activates GSDMD and induces pore formation and pyroptosis [116].

### Physiological consequences of GSDM localization on endosomal and lysosomal membranes

Although information about GSDM physiological roles in endosome‐lysosome biology remains scarce, these organelles have a long‐standing link to pyroptosis. Lysosomes contain antimicrobial proteins that remain active even after exocytosis, enhancing bacterial clearance in the extracellular environment. During infection, caspase‐1 activation and PM permeabilization increase intracellular calcium levels, promoting lysosome exocytosis and the release of host antimicrobial factors and microbial products, thereby boosting the antimicrobial activity of pyroptotic cell supernatant [140]. Similarly, lysosomes contribute to the repair of GSDM‐induced PM damage. Upon GSDMD pore formation, Ca^2+^ influx triggers lysosomal exocytosis, leading to the release of ASM. Caspase‐7‐mediated processing of ASM promotes sphingomyelin conversion to ceramide, which initiates membrane repair [130] (see also ‘GSDM localization at the plasma membrane’).

Importantly, a direct functional role of GSDM‐mediated lysosomal permeabilization has recently emerged. During mitochondrial anchored protein ligase (MAPL)‐induced cell death, mitochondrial‐derived vesicles (MDVs) containing mtDNA are delivered to lysosomes, where fusion occurs, resulting in mitochondria‐lysosome‐related organelles (MLROs). Concurrently, GSDMs are activated, leading to lysosomal permeabilization and the subsequent release of mtDNA into the cytosol. This process activates the cGAS‐STING pathway, linking mitochondrial stress to immune signaling [117].

### GSDM localization at other organelles and physiological consequences

Emerging evidence suggests that GSDM localization is extended even to the nucleus and the endoplasmic reticulum [47]. In the nucleus, GSDMs exhibit noncanonical functions, acting as important transcription modulators. To date, GSDMB and GSDMD have been shown to accumulate in the nucleus of human bronchial epithelial cells and colorectal cancer cells, respectively. In human bronchial epithelial cells, overexpression of GSDMB isoform 1 upregulates TGF‐β1 through a mechanism that requires GSDMB nuclear localization [39]. GSDMB‐1 also localizes to the nucleus of the human breast cancer cell line MCF7 and the human cervical cancer cell line HeLa, whereas it is exclusively found in the cytoplasm of the hepatocellular carcinoma cell line HepG2 [38]. In colorectal cancer cells, GSDMD regulates its subcellular distribution from the cytoplasm to the nucleus where it interacts with the ADP‐ribosylation factor PARP‐1 [Poly (ADP‐ribose) polymerase 1] to dramatically inhibit its DNA damage repair function [40]. GSDMD nuclear localization has also been detected in other cell types. In the intestinal epithelial cells (IEC), a 13 kDa N‐terminal GSDMD fragment, rather than the canonical 30 kDa GSDMD executing pyroptosis, accumulates and translocates to the nucleus to induce the transcription of CIITA and major histocompatibility complex class II (MHCII) molecules. These molecules, in turn, induce Type 1 regulatory (Tr1) T cells in the upper small intestine to maintain immune tolerance to food [42]. In hepatocytes, GSDMD acts as a transcriptional regulator initiating the activation of transcription factor Stat5a, which subsequently facilitates the expression of CXCL1 [43]. GSDME also localizes to the nucleus of pancreatic ductal adenocarcinoma (PDAC) tumor cells to act both as a transcriptional regulator and as a transporter to mediate the entry of the transcription factor Y‐box‐binding protein 1 (YBX1) and promote mucin expression [41]. In keratinocytes, GSDME promotes the translocation of p65 into the nucleus for the progression of skin inflammation [44]. Chicken GSDMA‐NT also strongly localizes to and forms puncta in the nucleus, suggesting some evolutionary roles for GSDMA nuclear localization [45].

In addition to GSDM nuclear localization, a relevant role of PJVK at peroxisomes has been characterized. PJVK localizes at the peroxisomal membrane and is essential for both the proliferation of peroxisomes and the degradation of damaged peroxisomes through selective autophagy caused by oxidative stress due to sound overstimulation. Indeed, mice deficient in PJVK exhibit increased susceptibility to noise‐induced oxidative stress, which is attributed to peroxisomal dysfunction and insufficient antioxidant defenses in the cochlea [125, 126].

Roles for GSDMD in ER and extracellular vesicles are also emerging. In HepG2 cells, a model of liver carcinoma, when ER stress is stimulated, the ER stress sensor inositol‐requiring enzyme 1α (IRE‐1α) has been identified as the ER‐derived modulator of pyroptosis via direct binding and activation of GSDMD [37]. Conversely, GSDMD has also been shown to trigger ER stress. In cardiomyocytes treated with doxorubicin, GSDMD forms pores on the ER membrane, stimulating ER fragmentation via FAM134B, an ER‐phagy receptor, and ultimately promoting autophagy and apoptosis [36]. Similarly, during cisplatin chemotherapy, GSDMD overexpression has been found to increase phosphorylation of eukaryotic translation initiation factor 2 alpha (eIF2α), activating the ER stress response and promoting tumor cell apoptosis rather than pyroptosis [118]. Furthermore, GSDMD targeting the ER is responsible for calcium leakage, which in turn facilitates the translocation of HMGB1 from the nucleus to the cytosol, rendering this DAMP available for release in sepsis [35].

GSDMD plays also important roles in secretory vesicles both as inactive full‐length or active pore‐forming protein. In colonic IECs, GSDMD FL, chaperoned by Cdc37/Hsp90, recruits the E3 ligase NEDD4 to catalyze polyubiquitination of pro–IL‐1β and facilitates its release via small extracellular vesicles (sEVs) in response to caspase‐8 inflammasome activation. This GSDMD‐guided IL‐1β release is crucial for the development of intestinal inflammation [120]. On the contrary, GSDMD‐containing extracellular vesicles, released during pyroptosis, mediate the dissemination of GSDMD pores to neighboring cells through cell‐to‐cell vesicular transfer, enhancing the diffusion of cell death [119].

## Pathological implications of GSDM cellular membrane alterations in infection

Since their discovery, GSDMs have garnered significant interest for their importance in various pathologies encompassing hearing impairment [17], asthma [155], and hair loss [156]. In recent years, they have also emerged as promising therapeutic targets for infections and cancer.

GSDM activation in the context of infections has been the focus of extensive research [6, 157]. PM pore formation not only restricts the spread of pathogens by inducing cell death in infected cells but also promotes the release of pro‐inflammatory cytokines that function as DAMPs. Excessive cytokine secretion triggers a broad range of inflammatory responses, including the self‐sustained activation of immune cells and hyperinflammation, which can lead to life‐threatening conditions [136, 137, 158]. An unrestrained inflammatory response to infection is indeed responsible for sepsis and related disseminated intravascular coagulation (DIC) and acute organ dysfunction, including acute respiratory distress syndrome (ARDS) and acute kidney injury (AKI) [159, 160, 161, 162]. Sepsis, despite several immunotherapy trials, remains a major cause of morbidity and mortality [162]. In sepsis, various DAMPs, such as HMGB1, eCIRP, and heat shock proteins serve as signals for the inflammatory response. Targeting GSDMD can serve as a novel therapeutic strategy for sepsis [163]. Targeted knockdown of GSDMD in hepatocytes has been shown to improve survival in septic mice and reduce overall hyperinflammation by impairing pore formation at the ER and consequent HMGB1 release [35].

## Pathological implications of GSDM cellular membrane alterations in cancer

Emerging research has also highlighted a role for GSDMs in cancer biology, revealing both diverse and contrasting functions. These proteins exhibit a range of cell death‐dependent and death‐independent activities, showcasing either pro‐tumor or anti‐tumor properties depending on GSDM types and cellular context, and influencing sensitization to or resistance against oncologic treatments.

GSDMB overexpression in various cancers, including hepatocellular carcinoma, gastric, breast, and cervix cancers has been mainly associated with tumor progression. The upregulation of endogenous GSDMB isoform 2 correlates with decreased survival rates and enhanced tumor growth and metastasis in breast cancer patients [164] contributing to resistance against anti‐HER2 therapies through prosurvival autophagy mechanisms [165]. Opposing its pro‐tumor functions, GSDMB can exhibit pyroptotic activity and an anti‐tumor effect in the context of an activated anti‐tumor immune response, where its activation, mediated by GZMA from cytotoxic lymphocytes, promotes tumor clearance [12].

Whereas the role of GSDMD in infection is well established, its involvement in cancer remains contentious. Different studies have reported both upregulation and downregulation of GSDMD in tumors [166]. GSDMD protein levels are significantly upregulated in nonsmall cell lung cancer (NSCLC) when compared to matched adjacent tumor specimens. Increased GSDMD expression has been associated with aggressive tumor characteristics, including larger tumor size and more advanced tumor‐node‐metastasis (TNM) stages [167]. Conversely, GSDMD acts as a tumor suppressor gene in colorectal and breast cancers [168]. Specifically, nuclear localization of GSDMD is associated with favorable clinical outcomes in colorectal cancer [169].

Among all GSDMs, GSDME has the most well‐defined role in cancer and is the most extensively investigated in anti‐tumor therapies [166, 170]. In most human cancer cell lines, GSDME is epigenetically suppressed by promoter DNA methylation, and the majority of tumor‐associated GSDME mutations studied impair its ability to induce pyroptosis [28, 171]. Additionally, GSDME expression in cancer models generally inhibits cell growth and induces cell death, while various antioncologic treatments have been shown to induce GSDME processing and cell death [15, 89, 172, 173, 174].

Evading apoptosis is a cancer hallmark that can provide resistance to anticancer therapies. In this scenario, activating pyroptosis or other cell death mechanisms could lead to tumor regression, and GSDME activation by apoptotic caspase‐3 facilitates a significant switch from an immunologically silent apoptotic response to an inflammatory, immunologically active pyroptotic state in cancer cells.

Therefore, inducing GSDM activation in certain cancer cells can represent a promising therapeutic strategy. GSDM‐mediated PM pore formation in tumor cells can promote the release of inflammatory cytokines, transforming the tumor immune microenvironment (TIME) from a ‘cold’ (noninflamed) to a ‘hot’ (inflamed) state. Thus, in addition to directly killing cancer cells, GSDMs may exert a secondary effect by enhancing tumor immunotherapy. GSDME activation can modulate infiltration of immune cells, including tumor‐associated macrophages (TAMs), through EIF2AK2, significantly improving anti‐tumor immunotherapy [28, 175]. Immune cell recruitment upon GSDME activation is indeed fundamental to expanding several innovative therapeutic strategies, such as anti‐PD1 and CAR T‐cell treatment [176, 177]. For example, in oncolytic virus therapy, natural or engineered oncolytic viruses are used to activate GSDME for recruiting cytotoxic T lymphocytes, thereby augmenting the effectiveness of anti‐PD1 therapy [177].

In addition to GSDME, GSDMB and GSDMD have also been explored for their potential use in immunotherapy. A nucleus‐targeted nanoparticle (NP) platform has recently been developed for the systemic delivery of a plasmid expressing the N‐terminal domain of GSDMD for the treatment of oral squamous cell carcinoma (OSCC). This delivery system was designed not only to induce pyroptosis in OSCC cells but also to promote the secretion of functional chemokines and cytokines to boost NK cell‐based immunotherapy [178]. Additionally, inducing pyroptosis in cancer cells expressing pyroptosis‐activable GSDMB variants could represent a promising therapeutic strategy. Exogenous overexpression of GSDMB isoform‐3 in murine cancer cells influences immune stimulation with anti PD‐1 immunotherapy, leading to T‐cell/GZMA‐dependent tumor suppression [12]. The delivery of anti‐GSDMB antibodies using nanocapsules in the treatment of breast cancer enhances the binding of GSDMB to sulfatides. This interaction subsequently reduces cell migratory behavior and may upregulate the intrinsic cell death activity associated with GSDMB [179]. Moreover, inflammation has been shown to further enhance GSDMB expression in tumors, creating a positive feedback loop that can promote tumor cell death [12, 63]. Importantly, tumor cells often co‐express various cytotoxic and noncytotoxic GSDMB variants simultaneously. The presence of noncytotoxic isoforms can potentially interfere with the anti‐tumor effects of cytotoxic variants. The balance among these isoforms, along with their distinct pro‐ and anti‐tumor functions, may play a critical role in determining the clinical behavior of tumors and can be exploited for controlling pyroptotic side effects [99].

However, the activation of GSDMs in tumors can be detrimental. Prolonged exposure to the inflamed TIME can stimulate tumor cell proliferation and metastasis [180]. Immunotherapy strategies may lead to severe complications, such as the development of cytokine release syndrome (CRS), as a consequence of extensive and uncontrolled induction of pyroptosis [181, 182]. Furthermore, GSDME is abundantly expressed in both normal tissue cells and tumor‐infiltrating macrophages, which can exacerbate the toxicity and side effects associated with chemotherapy [15]. Recent studies have identified tumor‐targeting nanomaterials, photodynamic therapy, and aptamer‐conjugated outer membrane vesicles (Apt‐OMVs) as promising strategies to address these challenges by precisely targeting cancer cells [183].

## Conclusions

Originally identified for their role in pyroptosis, GSDMs have gained significant attention due to their broader implications in various pathophysiological conditions, largely driven by their ability to target different cellular membranes. The pleiotropic nature of GSDMs suggests context‐dependent regulation, where factors, such as stimulus intensity, cleavage site selection, subcellular localization, and cellular/tissue microenvironment, dictate distinct functional outcomes.

For example, distinct cleavage sites by different proteases generate GSDMD fragments with varying biological activities, influencing whether they drive pyroptosis, apoptosis, or nonlytic signaling functions [42, 46, 107, 116]. However, the mechanisms determining how distinct cleavage patterns translate into different GSDMD activities remain unclear.

Beyond cleavage, specific membrane targeting is a key determinant of GSDM function. While phospholipid binding is essential, it is not sufficient to explain specificity. The presence of certain lipids, such as PIP_2_, in different organelles raises the question of what are, besides membrane composition, the regulatory mechanisms determining a different subcellular membrane distribution. GSDM recruitment to a particular cellular compartment can be biased by a stimulus‐dependent regulation that could involve localized lipid metabolism, organelle stress signals, or scaffolding proteins. Additionally, lipid chaperones and lipidation events have been implicated in guiding GSDM membrane specificity, but their precise role requires further investigation. Furthermore, it remains unclear whether regulatory mechanisms governing pore formation, such as phosphoinositide metabolism, which is crucial for GSDMD at the PM, are conserved across all GSDMs.

Mitochondrial targeting of GSDMs is particularly intriguing, as BAX/BAK already mediate outer membrane permeabilization during apoptosis. Whether GSDM pores cooperate with, complement, or bypass apoptotic pores remains an open question. Additionally, do GSDM pores in mitochondria differ mechanistically and structurally from those formed at the PM or other organelles? Variations in pore size, stability, or composition may influence their ability to release specific molecules, thereby modulating pro‐inflammatory and cell death signals.

Another layer of complexity is whether different GSDMs, when targeting the same membrane, form functionally distinct pores. For instance, mitochondrial GSDMD pores lead to caspase‐3‐dependent activation of GSDME, which subsequently targets mitochondrial membranes [32, 34]. This raises questions about the role of this interplay between GSDMD and GSDME in mitochondrial dysfunction.

The factors that determine whether a GSDM‐mediated event is lytic or nonlytic remain poorly understood. In epithelial cells, GSDM pores allow cytokine or mucin release without causing cell death, while in immune cells, they drive inflammatory pyroptosis. Do variations in stimuli, activation, protein modifications, or pore structure define these functional differences? Understanding the molecular basis of these divergent outcomes is essential for targeted therapeutic modulation of GSDM activity.

GSDMs are also emerging as key players in cancer biology. Their ability to trigger pyroptosis can convert immunologically ‘cold’ tumors into ‘hot’ ones, enhancing anti‐tumor immunity. However, precisely controlling GSDM activation to selectively kill malignant cells while sparing normal tissue remains a major challenge.

Developing interventions that preserve physiological GSDM roles while preventing pathological hyperactivation is critical for translating these findings into clinical applications. To date, covalent inhibitors have been identified for GSDMD and predominantly focus on Cys191, but these inhibitors lack specificity [85, 86]. Alternative cysteine residues or regulatory pathways could provide novel therapeutic windows for fine‐tuning GSDM function while minimizing off‐target effects.

Moving forward, a systematic investigation into the regulatory networks governing GSDM activation, membrane targeting, pore structure, and functional outcomes is essential. Unraveling these complexities will not only deepen our understanding of cell death and immune signaling but also pave the way for precision‐targeted therapies in inflammation, infection, and cancer.

## Conflict of interest

The authors declare no conflict of interest.

## Author contributions

EM contributed to the conceptualization and writing – original draft preparation. NG contributed to the writing – original draft preparation. KC contribute to the conceptualization, resources, writing – original draft, supervision, project administration, and funding acquisition.
